# Economic Disparities in Utilization and Outcomes of Structural Heart Disease Interventions in the United States

**DOI:** 10.1016/j.jacadv.2024.101034

**Published:** 2024-07-03

**Authors:** Mahmoud Ismayl, Hasaan Ahmed, Andrew M. Goldsweig, Mackram F. Eleid, Mayra Guerrero

**Affiliations:** a Department of Cardiovascular Medicine, Mayo Clinic, Rochester, Minnesota, USA; b Department of Internal Medicine, Creighton University School of Medicine, Omaha, Nebraska, USA; c Department of Cardiovascular Medicine, Baystate Medical Center, Springfield, Massachusetts, USA

**Keywords:** economic disparities, LAAO, structural heart disease, TAVR, TMVr, TMVR

## Abstract

**Background:**

Disparities in access to care cause negative health consequences for underserved populations. Economic disparities in structural heart disease (SHD) interventions are not well characterized.

**Objectives:**

The objective of this study was to evaluate economic disparities in the utilization and outcomes of SHD interventions in the United States.

**Methods:**

We queried the National Inpatient Sample (2016-2020) to examine economic disparities in the utilization, in-hospital outcomes, length of stay, and cost of SHD interventions among patients ≥65 years of age. Outcomes were determined using logistic regression models.

**Results:**

A total of 401,005 weighted hospitalizations for transcatheter aortic valve replacement, left atrial appendage occlusion, transcatheter mitral valve repair, and transcatheter mitral valve replacement were included. Utilization rates (number of procedures performed per 100,000 hospitalizations) were higher in patients with high income compared with medium and low income for transcatheter aortic valve replacement (559 vs 456 vs 338), left atrial appendage occlusion (148 vs 136 vs 99), transcatheter mitral valve repair (65 vs 54 vs 41), and transcatheter mitral valve replacement (7.7 vs 6.7 vs 1.2) (all *P* < 0.01). Low- and medium-income patients had distinctive demographic and clinical risk profiles compared with high-income patients. There were no significant differences in the adjusted in-hospital mortality, key complications, or length of stay between high-, medium-, and low-income patients following any of the 4 SHD interventions. High-income patients incurred a modestly higher cost with any of the 4 SHD interventions compared with medium- and low-income patients.

**Conclusions:**

Economic disparities exist in the utilization of SHD interventions in the United States. Nonetheless, adjusted in-hospital outcomes were comparable among high-, medium-, and low-income patients. Multifaceted implementation strategies are needed to attenuate these utilization disparities.

Structural heart disease (SHD) represents a spectrum of noncoronary conditions associated with significant morbidity and mortality.^1^ Catheter-based SHD interventions, encompassing transcatheter aortic valve replacement (TAVR), left atrial appendage occlusion (LAAO), and transcatheter mitral valve repair (TMVr) and replacement (TMVR) have increasingly replaced surgery for SHD.^2^ Driven by technological advances in devices, imaging, and techniques, SHD interventions have grown rapidly and improve outcomes among individuals at all levels of surgical risk, even those once considered inoperable.^2^, ^3^, ^4^

Some inequities in access to SHD interventions are well-documented.^5^ While prior studies have evaluated racial and ethnic disparities in SHD interventions,^6^^,^^7^ data on the impact of economic disparities on the utilization and outcomes of SHD interventions remain scarce. Therefore, we queried the National Inpatient Sample (NIS) database to investigate economic disparities in the utilization and outcomes of common SHD interventions.

## Methods

### Data source and ethics statement

Hospitalization data were abstracted from the NIS database, which is part of the Healthcare Cost and Utilization Project (HCUP) family of databases sponsored by the Agency for Healthcare Research and Quality.^8^ The NIS is the largest publicly available, fully deidentified, all-payer inpatient healthcare database in the United States. The NIS is derived from billing data submitted by hospitals to statewide organizations across the United States and has reliable and verified patient linkage numbers that can be used to track patients across hospitals within each state while adhering to strict privacy guidelines. The NIS database contains both patient- and hospital-level information from approximately 1,000 hospitals and represents approximately 20% of all U.S. hospitalizations, covering >7 million unweighted hospitalizations each year. When weighted, the NIS extrapolates to the national level, representing 35 million hospitalizations each year. Up to 40 discharge diagnoses and 25 procedure codes are collected for each patient using International Classification of Diseases-10th Revision codes.^9^ The NIS is compiled annually, which allows for analysis of procedural trends over time.^10^ This study was exempt from the requirements of the Mayo Clinic Institutional Review Board because the NIS is a publicly available database composed of deidentified data.

### Study sample and patient selection

**Figure 1:**
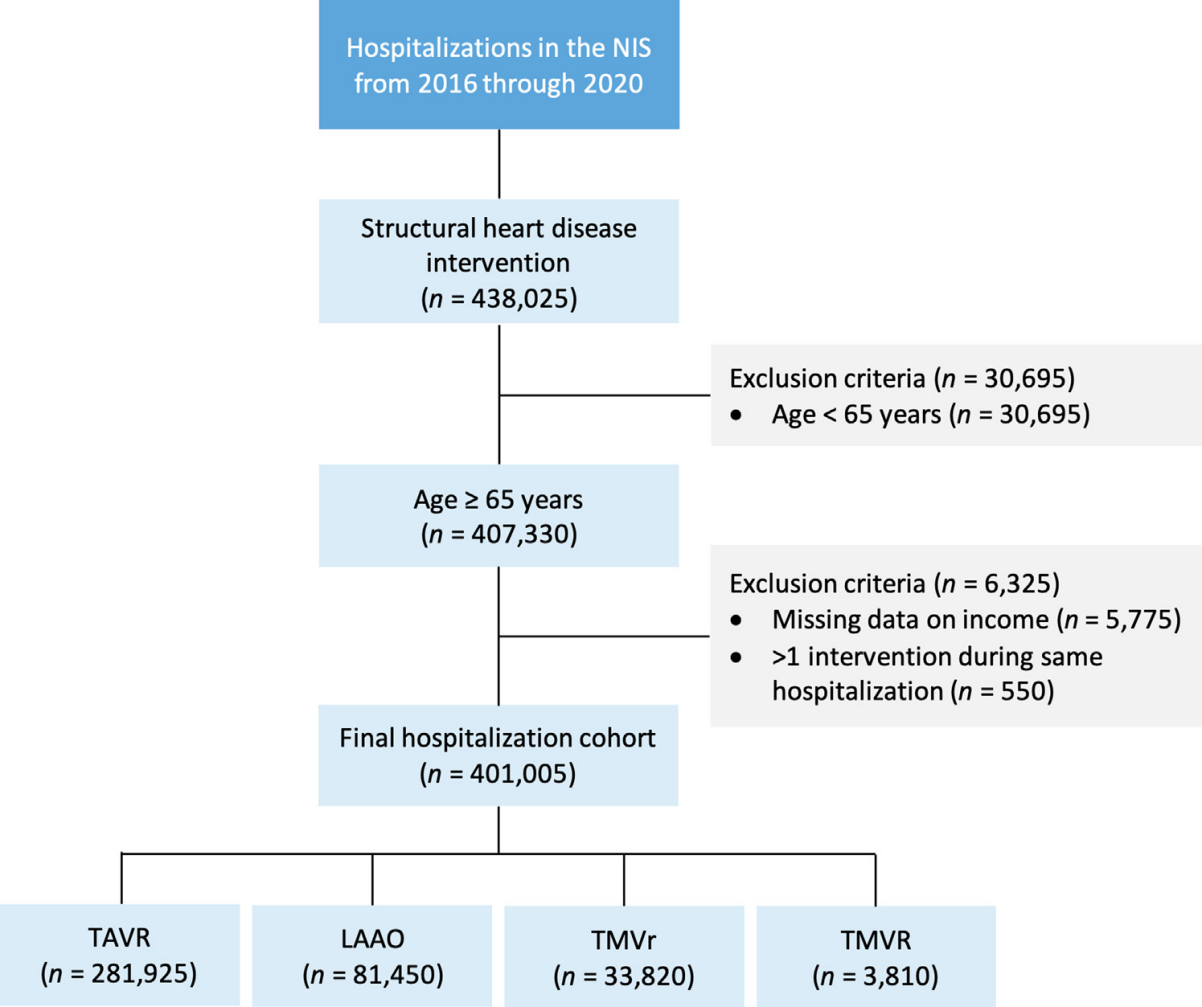
**Study Flow Diagram Showing Inclusion and Exclusion Criteria** Hospitalization counts represent national-level estimates. LAAO = left atrial appendage occlusion; NIS = National Inpatient Sample; TAVR = transcatheter aortic valve replacement; TMVr = transcatheter mitral valve repair; TMVR = transcatheter mitral valve replacement.

We queried the NIS database from January 2016 through December 2020 to identify hospitalizations in which patients ≥65 years of age underwent TAVR, percutaneous LAAO, TMVr, or TMVR using International Classification of Diseases-10th Revision, Procedure Coding System codes (02RF37H, 02RF38H, 02RF3JH, 02RF3KH, X2RF332, 02RF37Z, 02RF38Z, 02RF3JZ, 02RF3KZ for TAVR; 02L73DK for percutaneous LAAO; 02UG3JH, 02UG3JZ, 02QG3ZE, 02QG3ZZ for TMVr; and 02RG37H, 02RG38H, 02RG3JH, 02RG3KH, 02RG37Z, 02RG38Z, 02RG3JZ, 02RG3KZ for TMVR). A complete list of International Classification of Diseases-10th Revision diagnosis and procedure codes used in this study is presented in Supplemental Table 1. We excluded hospitalizations in which the patient was aged <65 years as well as those with missing data on income (Figure 1). We selected age 65 as a cutoff to minimize the potential confounding issue of lack of insurance coverage, given that most patients ≥65 years of age are eligible for the Centers of Medicare and Medicaid Services. Hospitalizations with >1 SHD intervention during the same admission were excluded from the outcomes analysis but not from the utilization and trend analyses.

The estimated median household incomes are ZIP code–specific, updated annually, and classified into 4 quartiles indicating the wealthiest to poorest populations. Similar to prior NIS studies, for hospitalizations that met inclusion criteria, we stratified the patients into 3 cohorts based on income: high income (75th-100th percentile), medium income (25th-75th percentile), and low income (0th-25th percentile).^11^ A more detailed explanation of all the variables in the NIS, including the specific dollar amounts in each category of median household income, is available on the HCUP website (https://www.hcup-us.ahrq.gov/db/nation/nis/nisdde.jsp).

### Study outcomes

The primary outcome was procedure utilization rate per income group defined as the number of procedures performed per 100,000 hospitalizations in patients aged ≥65 years. Secondary outcomes included temporal trends in SHD interventions in different income groups across the study period, as well as in-hospital complications including major adverse cardiovascular events (defined as a composite of all-cause in-hospital mortality or stroke), all-cause in-hospital mortality, stroke, acute kidney injury, major bleeding, vascular complications (defined as a composite of arteriovenous fistula, aneurysm, hematoma, retroperitoneal bleeding, or venous thromboembolism), permanent pacemaker placement, and cardiac tamponade. We also evaluated hospital length of stay (LOS), total hospital costs (inflation-adjusted to 2020 U.S. dollars^12^), and discharge disposition. Charge-to-cost ratio files were used to convert charges to costs at the individual hospital level.

### Statistical analysis

Descriptive statistics were presented as percentages for categorical variables and as median (IQR) for continuous variables. Categorical variables were compared using the Pearson chi-square test or Fisher exact test as appropriate. Continuous variables were compared using the Kruskal-Wallis 1-way analysis of variance.

A multivariable logistic regression analysis was constructed to adjust for potential confounders including age, sex, race, insurance, hospital location and teaching status, bed size, region, type of admission (elective/nonelective and weekend/weekday), Elixhauser and Charlson comorbidity index scores, and relevant comorbidities (Supplemental Table 2). Adjustment variables were selected a priori on the basis of their clinical significance, which may directly influence in-hospital outcomes. The results from these models are presented as adjusted ORs (aORs) with 95% CIs. Race was evaluated as an effect modifier for both utilization and outcomes of SHD interventions, and the results are presented separately for White and non-White patients along with the respective interaction *P* value. Trend analyses from 2016 through 2020 were conducted using linear regression.

In accordance with the HCUP data use agreement, we did not report variables that contained a small number of observed (ie, unweighted) hospitalizations (<11) as this could pose risks of subject identification and data privacy violation.^13^ A 2-tailed *P* < 0.05 was considered statistically significant. All statistical analyses were performed using Stata, version 17 (StataCorp) software, accounting for the NIS sampling design, and were weighted using sampling weights provided with the NIS database to estimate national-level effects per HCUP-NIS recommendations.^10^

## Results

### Utilization rate of SHD interventions per income group

**Figure 2:**
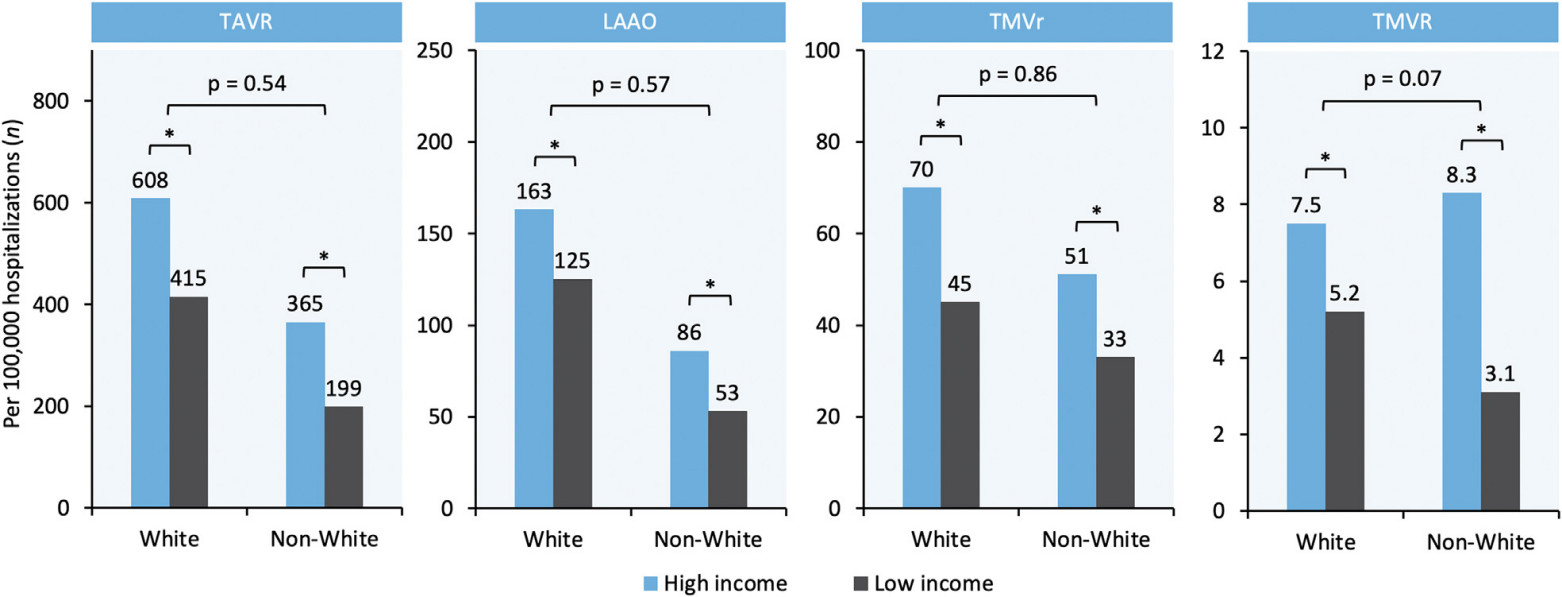
**Economic Disparities in the Utilization of SHD Interventions in Patients Aged ≥65 Years in the U.S. Stratified by Race** All interaction *P* > 0.05, indicating that race did not significantly modify the effect of income on utilization of SHD Interventions. ∗*P* < 0.01. LAAO = left atrial appendage occlusion; TAVR = transcatheter aortic valve replacement; TMVr = transcatheter mitral valve repair; TMVR = transcatheter mitral valve replacement.

A total of 401,005 weighted hospitalizations for SHD interventions were identified for analysis. In the overall cohort, patients in the high-income group constituted 26.7%, 24.4%, 26.5%, and 26.3% of all patients who underwent TAVR, LAAO, TMVr, and TMVR, respectively. The utilization rates (number of procedures/100,000 hospitalizations in patients aged ≥65 years) were significantly higher in patients with high income compared with medium and low income for all 4 procedures: TAVR (559 vs 456 vs 338), LAAO (148 vs 136 vs 99), TMVr (65 vs 54 vs 41), and TMVR (7.7 vs 6.7 vs 1.2) (all *P* < 0.01; Central Illustration). Race did not significantly modify the effect of income on utilization of SHD interventions (all interaction *P* > 0.05) (Figure 2).

**Central Illustration:**
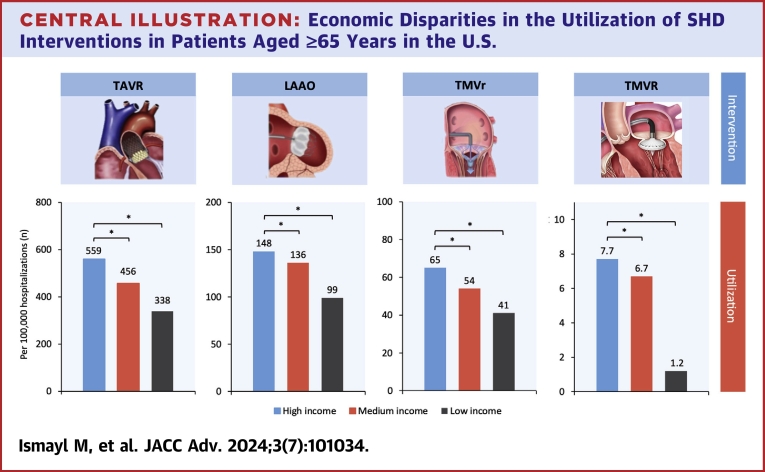
**Economic Disparities in the Utilization of SHD Interventions in Patients Aged ≥65 Years in the U.S.** ∗*P* < 0.01. LAAO = left atrial appendage occlusion; SHD = structural heart disease; TAVR = transcatheter aortic valve replacement; TMVr = transcatheter mitral valve repair; TMVR = transcatheter mitral valve replacement.

In multivariable regression analysis, low income was independently associated with lower TAVR use (aOR: 0.93, 95% CI: 0.88-0.98) and TMVr use (aOR: 0.89, 95% CI: 0.81-0.98) compared with high income. Medium income was independently associated with lower TAVR use (aOR: 0.95, 95% CI: 0.91-0.99) compared with high income.

### Temporal trends in SHD interventions

**Figure 3:**
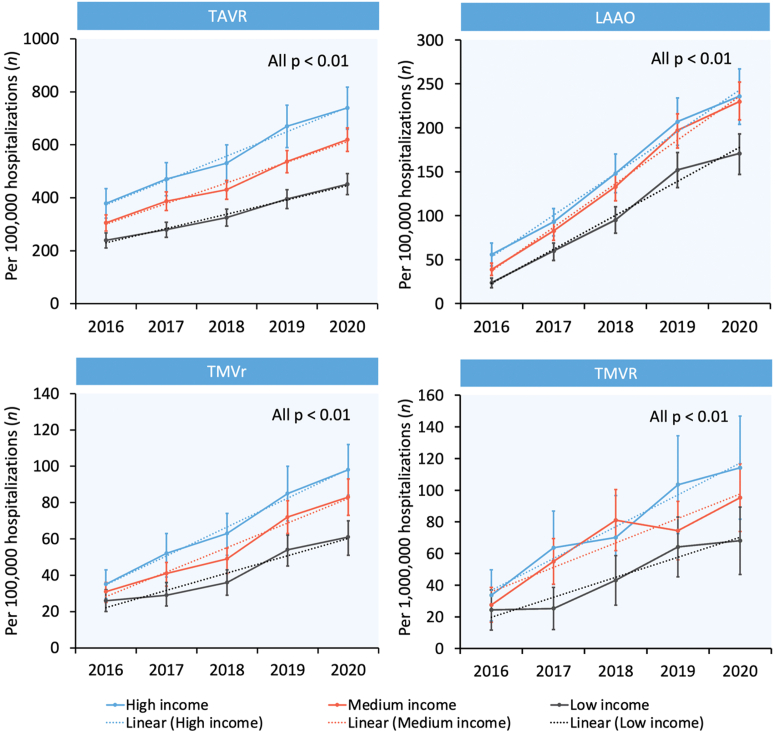
**Year-Over-Year Trend in the Number of SHD Interventions Performed in Patients Aged ≥65 Years in the U.S. Stratified by Economic Status** LAAO = left atrial appendage occlusion; TAVR = transcatheter aortic valve replacement; TMVr = transcatheter mitral valve repair; TMVR = transcatheter mitral valve replacement.

From 2016 through 2020, the use of TAVR increased significantly in all 3 income groups (379 to 739 [high income], 306 to 620 [medium income], and 239 to 451 [low income] per 100,000 hospitalizations in patients aged ≥65 years, all *P*_trend_ <0.01). The use of LAAO increased significantly in all 3 income groups (56 to 236 [high income], 39 to 230 [medium income], and 24 to 171 [low income] per 100,000 hospitalizations in patients aged ≥65 years, all *P*_trend_ <0.01). Similarly, the use of TMVr increased significantly in all 3 income groups (35 to 98 [high income], 31 to 83 [medium income], and 26 to 61 [low income] per 100,000 hospitalizations in patients aged ≥65 years, all *P*_trend_ <0.01). Finally, the use of TMVR increased significantly in all 3 income groups (34 to 114 [high income], 28 to 95 [medium income], and 24 to 68 [low income] per 1,000,000 hospitalizations in patients aged ≥65 years, all *P*_trend_ <0.01). Annual trends for SHD interventions stratified by income group are shown in Figure 3.

### Baseline risk profile among income groups

There were notable differences in demographic, hospital, and clinical characteristics among patients undergoing SHD interventions across the different income groups. Low-income patients were younger and more likely to be female, Black or Hispanic, and receive care in rural hospitals with small bed sizes compared with high-income patients. Regional variations were also observed, with low-income patients undergoing SHD interventions more commonly in the South and Midwest and less commonly in the Northeast and West compared with high-income patients.

Low-income patients undergoing TAVR, LAAO, and TMVr had higher Elixhauser and Charlson comorbidity index scores compared with high-income patients, mainly driven by the higher rates of diabetes mellitus, obesity, coronary artery disease, congestive heart failure, dialysis dependence, and chronic pulmonary disease. High-income patients were more likely to have cancer. Baseline characteristics stratified by income group are shown in Tables 1 and 2.

**Table 1:** Baseline Characteristics of Patients Undergoing TAVR and LAAO Stratified by Income

	TAVR				LAAO			
	High Income (n = 75,245)	Medium Income (n = 147,770)	Low Income (n = 58,910)	*P* Value	High Income (n = 19,900)	Medium Income (n = 44,115)	Low Income (n = 17,435)	*P* Value
Demographic characteristics								
Age, y	82 (76-87)	81 (75-86)	80 (74-85)	<0.01	78 (73-83)	77 (72-82)	77 (72-82)	<0.01
65–74	20.4	23.0	26.1	<0.01	31.7	34.6	37.4	<0.01
75–84	44.4	46.0	46.2		50.7	50.2	48.7	
85+	35.2	31.0	27.7		17.6	15.2	13.9	
Biological sex								
Male	56.7	55.1	52.4	<0.01	62.2	57.4	52.5	<0.01
Female	43.3	44.9	47.6		37.8	42.6	47.5	
Race/ethnicity								
White	89.1	89.7	81.3	<0.01	90.6	89.9	83.5	<0.01
Black	2.0	2.7	8.8		1.8	3.0	7.8	
Hispanic	3.3	4.2	7.2		3.0	4.3	6.1	
Other^a^	5.6	3.4	2.7		4.6	2.8	2.6	
Insurance								
Medicare	93.0	93.8	93.1	0.02	93.4	94.1	93.5	0.33
Medicaid	0.5	0.4	0.7		0.3	0.3	0.5	
Private insurance	6.2	5.5	5.9		5.8	5.2	5.6	
Self-pay	0.3	0.3	0.3		0.5	0.4	0.4	
Hospital characteristics								
Location/teaching status								
Rural	0.2	1.3	2.3	<0.01	0.2	2.2	4.5	<0.01
Urban nonteaching	7.4	10.0	9.9		10.2	10.7	9.8	
Urban teaching	92.4	88.7	87.8		89.6	87.1	85.7	
Bed size^b^								
Small	6.1	7.3	8.4	0.01	8.8	10.8	11.7	0.16
Medium	23.1	20.4	21.5		24.5	23.3	25.1	
Large	70.8	72.3	70.1		66.7	65.9	63.2	
Region								
Northeast	37.2	20.5	11.4	<0.01	26.4	14.5	9.5	<0.01
Midwest	16.2	27.1	21.6		18.1	25.7	19.7	
South	21.3	32.4	53.1		26.6	38.8	55.7	
West	25.3	20.0	13.9		28.9	21.0	15.1	
Elective admission	83.5	83.4	82.7	0.37	92.8	92.4	91.6	0.34
Weekend admission	3.5	3.1	3.3	0.15	0.8	0.9	0.8	0.81
Clinical characteristics								
Elixhauser comorbidity index	5 (4-7)	5 (4-7)	6 (4-7)	<0.01	4 (3-5)	4 (3-5)	4 (3-6)	<0.01
Charlson comorbidity index	3 (1-4)	3 (1-4)	3 (2-4)	<0.01	2 (1-3)	2 (1-3)	2 (1-3)	<0.01
0	8.4	6.5	5.6	<0.01	23.3	21.4	20.2	<0.01
1	20.6	18.9	17.8		26.4	23.6	22.9	
2	20.3	20.4	19.3		18.3	19.1	20.0	
≥3	50.7	54.2	57.3		32.0	35.9	36.9	
Diabetes mellitus	33.4	37.6	41.6	<0.01	30.8	34.6	38.2	<0.01
Hypertension	89.5	90.2	91.0	<0.01	86.1	86.7	89.0	<0.01
Dyslipidemia	75.0	73.7	72.3	<0.01	64.6	62.5	62.9	0.10
Nicotine/tobacco use	40.0	39.8	39.8	0.95	38.5	38.9	39.2	0.85
Alcohol abuse	1.4	1.1	1.0	0.02	1.4	1.3	1.2	0.77
Drug abuse	0.3	0.3	0.4	0.52	0.2	0.3	0.4	0.15
Obesity	16.8	19.7	21.1	<0.01	14.9	16.4	17.4	0.03
Coronary artery disease	67.9	69.8	70.8	<0.01	47.5	50.2	52.3	<0.01
Peripheral vascular disease	21.3	22.4	22.5	0.05	15.6	16.2	16.2	0.65
Congestive heart failure	70.8	73.8	74.5	<0.01	37.3	39.6	41.3	<0.01
Renal failure	33.1	34.7	35.4	<0.01	22.8	25.0	24.6	0.04
Dialysis dependent	2.3	2.2	2.8	<0.01	1.6	2.3	3.0	<0.01
Liver disease	3.3	2.9	3.1	0.09	2.2	2.4	2.8	0.16
Chronic pulmonary disease	23.7	27.4	30.3	<0.01	18.7	23.0	25.2	<0.01
Obstructive sleep apnea	13.6	14.1	13.6	0.20	17.7	17.5	16.7	0.47
Coagulopathy	12.3	10.8	10.5	<0.01	5.0	3.7	3.6	<0.01
Cancer	4.4	3.9	3.4	<0.01	3.0	2.2	1.6	<0.01
Malnutrition	1.6	1.6	1.7	0.49	0.3	0.2	0.1	0.42
Dementia	4.4	4.4	4.5	0.82	3.3	2.9	3.2	0.32
Depression	8.2	8.0	7.3	0.02	7.3	8.2	7.6	0.17
Previous history								
Myocardial infarction	11.4	12.5	12.8	<0.01	12.3	12.8	14.0	0.08
Stroke/TIA	11.6	11.8	12.1	0.51	23.4	22.7	24.2	0.18
Cardiac arrest	0.4	0.3	0.3	0.29	0.5	0.5	0.5	0.90
PCI	22.2	22.2	21.8	0.73	16.2	17.5	17.6	0.15
CABG	14.6	16.1	16.6	<0.01	13.8	14.5	14.5	0.51
ICD	2.3	2.4	2.6	0.27	6.4	6.1	6.6	0.63
PPM	9.6	9.5	9.6	0.80	18.4	18.4	19.8	0.23

CABG = coronary artery bypass grafting; ICD = implantable cardioverter-defibrillator; LAAO = left atrial appendage occlusion; PCI = percutaneous coronary intervention; PPM = permanent pacemaker; TAVR = transcatheter aortic valve replacement; TIA = transient ischemic attack.

Values are median (IQR) or %. Two authors (M.I. and H.A.) independently verified the International Classification of Diseases-10th Revision (ICD-10) codes that corresponded to each comorbidity (Supplemental Table 1), and any disagreements in inclusion or exclusion of ICD-10 codes were discussed with a third author (A.M.G).

a Other includes Asian or Pacific Islander, Native American, and Other.

b Bed-size categories are based on inpatient beds and are specific to the hospital’s location and teaching status.

**Table 2:** Baseline Characteristics of Patients Undergoing TMVr and TMVR Stratified by Income

	TMVr				TMVR			
	High Income (n = 8,950)	Medium Income (n = 17,725)	Low Income (n = 7,145)	*P* Value	High Income (n = 1,000)	Medium Income (n = 2,070)	Low Income (n = 740)	*P* Value
Demographic characteristics								
Age, y	82 (76-86)	81 (75-86)	79 (74-84)	<0.01	78 (74-83)	76 (71-82)	78 (73-83)	<0.01
65–74	18.5	22.0	29.0	<0.01	28.5	40.1	31.8	0.04
75–84	47.0	46.7	46.4		54.5	45.7	51.4	
85+	34.5	31.3	24.6		17.0	14.2	16.8	
Biological sex								
Male	54.4	54.2	50.4	0.03	45.0	43.2	44.6	0.90
Female	45.6	45.8	49.6		55.0	56.8	55.4	
Race/ethnicity								
White	86.3	85.3	73.1	<0.01	82.1	85.0	76.8	<0.01
Black	2.7	5.3	14.7		2.1	7.1	11.2	
Hispanic	3.5	5.5	7.8		6.3	3.3	7.4	
Other^a^	7.5	3.9	4.4		9.5	4.6	4.6	
Insurance								
Medicare	93.5	93.4	92.9	0.96	92.9	89.3	96.6	0.17
Medicaid	0.7	0.6	0.6		NR^b^	0.7	NR^b^	
Private insurance	5.5	5.8	6.2		5.6	9.3	2.7	
Self-pay	0.3	0.2	0.3		NR^b^	0.7	0	
Hospital characteristics								
Location/teaching status								
Rural	0.1	0.8	1.4	0.03	0	1.0	NR^b^	0.73
Urban nonteaching	8.4	7.7	8.3		5.5	5.6	6.8	
Urban teaching	91.5	91.5	90.3		94.5	93.4	92.6	
Bed size^c^								
Small	5.1	7.6	9.1	0.04	6.7	8.2	10.5	0.04
Medium	19.8	18.1	19.0		14.9	11.4	20.0	
Large	75.1	74.3	71.9		78.4	80.4	69.5	
Region								
Northeast	30.1	15.4	8.9	<0.01	35.5	16.4	9.5	<0.01
Midwest	16.5	23.6	17.2		15.5	26.8	25.0	
South	21.7	35.3	53.8		19.0	33.1	46.6	
West	31.7	25.7	20.1		30.0	23.7	18.9	
Elective admission	79.3	79.1	76.7	0.17	74.0	70.3	70.1	0.61
Weekend admission	3.9	3.4	4.3	0.33	10.0	8.2	5.4	0.30
Clinical characteristics								
Elixhauser comorbidity index	6 (4-7)	6 (4-7)	6 (5-7)	<0.01	6 (5-8)	6 (5-8)	6 (5-8)	0.88
Charlson comorbidity index	3 (1-4)	3 (1-4)	3 (2-5)	<0.01	3 (2-4)	3 (2-4)	3 (1-5)	0.95
0	6.4	5.1	3.6	<0.01	3.0	3.4	4.1	0.95
1	22.0	20.9	19.0		21.5	21.0	23.0	
2	19.6	18.6	17.5		18.0	19.8	21.5	
≥3	52.0	55.4	59.9		57.5	55.8	51.4	
Diabetes mellitus	22.3	27.7	30.8	<0.01	24.5	25.4	31.1	0.31
Hypertension	84.7	85.8	87.6	0.10	85.0	84.1	89.2	0.33
Dyslipidemia	64.3	62.9	62.9	0.60	62.5	63.8	63.5	0.96
Nicotine/tobacco use	37.0	37.6	38.0	0.86	31.5	32.9	34.5	0.84
Alcohol abuse	0.8	0.9	1.0	0.72	NR^b^	2.2	0	0.06
Drug abuse	0.3	0.5	0.6	0.41	NR^b^	NR^b^	NR^b^	-
Obesity	8.1	10.1	10.8	0.02	8.0	12.6	8.8	0.15
Coronary artery disease	63.5	63.2	67.6	0.01	63.0	64.5	66.9	0.76
Peripheral vascular disease	23.0	23.5	24.6	0.56	22.5	23.9	21.6	0.83
Congestive heart failure	85.5	86.2	89.5	<0.01	90.5	88.4	91.5	0.48
Renal failure	39.5	40.7	42.0	0.35	42.5	38.9	35.8	0.45
Dialysis dependent	2.1	2.8	3.6	0.03	2.5	2.4	NR^b^	0.72
Liver disease	3.7	3.0	3.4	0.33	4.0	7.2	4.7	0.23
Chronic pulmonary disease	23.9	25.7	32.7	<0.01	24.0	30.0	32.4	0.18
Obstructive sleep apnea	12.6	13.7	11.5	0.09	17.5	16.2	10.8	0.20
Coagulopathy	10.3	9.1	7.8	0.06	26.0	25.8	17.6	0.12
Cancer	3.8	3.0	2.0	0.02	3.0	2.7	NR^b^	0.59
Malnutrition	2.3	2.7	2.4	0.73	5.0	2.9	5.4	0.27
Dementia	4.1	3.4	2.6	0.06	1.5	1.9	NR^b^	0.87
Depression	7.8	7.9	7.1	0.64	10.5	9.2	8.8	0.84
Previous history								
Myocardial infarction	15.1	15.1	17.3	0.14	12.0	7.2	12.8	0.05
Stroke/TIA	10.7	10.3	11.0	0.77	11.0	12.8	12.8	0.80
Cardiac arrest	0.9	0.8	0.3	0.11	NR^b^	0.7	0	0.50
PCI	21.3	21.6	19.9	0.40	8.5	13.0	10.8	0.24
CABG	18.2	20.6	22.6	0.01	22.0	30.2	30.4	0.09
ICD	11.7	11.1	12.1	0.52	9.0	9.2	8.8	0.99
PPM	12.1	12.7	11.5	0.49	19.0	18.1	17.6	0.94

CABG = coronary artery bypass grafting; ICD = implantable cardioverter-defibrillator; PCI = percutaneous coronary intervention; PPM = permanent pacemaker; TIA = transient ischemic attack; TMVr = transcatheter mitral valve repair; TMVR = transcatheter mitral valve replacement.

Values are median (IQR) or %. Two authors (M.I. and H.A.) independently verified the International Classification of Diseases-10th Revision (ICD-10) codes that corresponded to each comorbidity (Supplemental Table 1), and any disagreements in inclusion or exclusion of ICD-10 codes were discussed with a third author (A.M.G).

a Other includes Asian or Pacific Islander, Native American, and Other.

b Cell counts <11 are not reportable (NR) per HCUP guidelines.

c Bed-size categories are based on inpatient beds and are specific to the hospital’s location and teaching status.

### In-hospital outcomes, LOS, and costs of SHD interventions stratified by income group

Despite the underutilization of SHD interventions in low- and medium-income groups, no significant differences in in-hospital outcomes were observed. With TAVR, there were no statistically significant differences in in-hospital mortality or key post-TAVR complications among the 3 income groups after adjustment for potential confounders. Similarly, in-hospital outcomes following LAAO, TMVr, and TMVR were similar across all income groups after adjustment for potential confounders. Although the hospital LOS associated with SHD interventions was similar among the 3 groups, the costs of all 4 procedures were significantly higher in high-income patients. In-hospital outcomes, LOS, and costs of SHD interventions stratified by income group are shown in Tables 3 and 4. Race did not significantly modify the effect of income on in-hospital outcomes, LOS, and costs of SHD interventions (all interaction *P* > 0.05) (Supplemental Tables 3 to 6).

**Table 3:** In-Hospital Outcomes of TAVR and LAAO Stratified by Income

	TAVR				LAAO			
	High Income (n = 75,245)	Medium Income (n = 147,770)	Low Income (n = 58,910)	*P* Value	High Income (n = 19,900)	Medium Income (n = 44,115)	Low Income (n = 17,435)	*P* Value
Complications								
MACE								
%	3.4	3.4	3.6	0.72	0.8	0.9	0.9	0.70
uOR (95% CI)	Ref.	0.99 (0.89-1.11)	1.04 (0.91-1.19)	–	Ref.	1.20 (0.79-1.81)	1.11 (0.67-1.81)	–
aOR (95% CI)^a^	Ref.	1.06 (0.94-1.21)	1.13 (0.97-1.33)	–	Ref.	1.28 (0.80-2.04)	1.05 (0.60-1.83)	–
Death								
%	1.2	1.3	1.4	0.37	0.1	0.2	0.2	0.23
uOR (95% CI)	Ref.	1.04 (0.87-1.25)	1.16 (0.94-1.43)	–	Ref.	2.41 (0.71-8.22)	3.05 (0.81-11.45)	–
aOR (95% CI)^a^	Ref.	1.12 (0.93-1.35)	1.24 (0.98-1.56)	–	Ref.	2.46 (0.68-8.84)	2.83 (0.68-11.80)	–
Stroke								
%	2.4	2.3	2.4	0.79	0.7	0.8	0.7	0.84
uOR (95% CI)	Ref.	0.97 (0.86-1.11)	1.02 (0.87-1.19)	–	Ref.	1.08 (0.70-1.69)	0.94 (0.54-1.64)	–
aOR (95% CI)^a^	Ref.	1.03 (0.88-1.21)	1.12 (0.92-1.37)	–	Ref.	1.13 (0.68-1.88)	0.84 (0.44-1.60)	–
Acute kidney injury								
%	9.4	9.7	10.7	**<0.01**	2.4	2.4	2.4	0.95
uOR (95% CI)	Ref.	1.04 (0.97-1.11)	**1.16 (1.06-1.26)**	–	Ref.	1.03 (0.80-1.32)	0.98 (0.73-1.33)	–
aOR (95% CI)^a^	Ref.	1.00 (0.93-1.09)	1.10 (0.99-1.22)	–	Ref.	0.92 (0.70-1.20)	0.83 (0.60-1.16)	–
Major bleeding								
%	14.4	14.8	14.9	0.56	6.0	6.2	6.3	0.81
uOR (95% CI)	Ref.	1.04 (0.96-1.12)	1.04 (0.95-1.15)	–	Ref.	1.05 (0.89-1.24)	1.06 (0.87-1.29)	–
aOR (95% CI)^a^	Ref.	1.02 (0.95-1.10)	0.99 (0.90-1.10)	–	Ref.	1.01 (0.85-1.19)	0.95 (0.76-1.17)	–
Vascular complications								
%	4.8	4.0	4.4	**<0.01**	1.0	1.2	1.3	0.48
uOR (95% CI)	Ref.	**0.83 (0.75-0.91)**	0.91 (0.81-1.02)	–	Ref.	1.23 (0.86-1.77)	1.26 (0.81-1.94)	–
aOR (95% CI)^a^	Ref.	0.93 (0.85-1.02)	0.97 (0.86-1.10)	–	Ref.	1.20 (0.83-1.72)	1.28 (0.80-2.05)	–
PPM placement								
%	8.8	8.4	7.3	**<0.01**	1.2	1.4	1.4	0.70
uOR (95% CI)	Ref.	0.94 (0.88-1.02)	**0.82 (0.74-0.90)**	–	Ref.	1.13 (0.82-1.57)	1.17 (0.78-1.74)	–
aOR (95% CI)^a^	Ref.	0.96 (0.89-1.04)	0.92 (0.83-1.01)	–	Ref.	1.07 (0.76-1.50)	1.08 (0.70-1.67)	–
Cardiac tamponade								
%	0.8	0.8	0.6	**0.03**	0.5	0.7	0.7	0.40
uOR (95% CI)	Ref.	0.94 (0.75-1.16)	**0.68 (0.50-0.92)**	–	Ref.	1.40 (0.85-2.31)	1.36 (0.77-2.42)	–
aOR (95% CI)^a^	Ref.	1.04 (0.82-1.31)	0.82 (0.60-1.13)	–	Ref.	1.36 (0.81-2.30)	1.30 (0.71-2.41)	–
Discharge disposition								
Routine	65.4	69.6	69.8	**<0.01**	92.8	92.4	91.9	0.47
Transfer to short–term hospital	0.3	0.3	0.3		NR^b^	0.1	0.1	
Transfer to SNF or ICF	11.2	10.8	11.2		2.2	2.5	2.5	
Home health care	21.8	17.9	17.2		4.9	4.8	5.2	
Resource utilization								
LOS (d)	2 (1-4)	2 (1-4)	2 (1-4)	0.21	1 (1-1)	1 (1-1)	1 (1-1)	0.71
Hospital cost ($)	56,408 (39,552-85,971)	52,180 (36,707-81,834)	50,461 (35,603-78,650)	**<0.01**	30,208 (21,667-46,187)	28,884 (20,882-41,942)	27,155 (19,412-39,322)	**<0.01**

aOR = adjusted odds ratio; ICF = intermediate care facility; LAAO = left atrial appendage occlusion; LOS = length of stay; MACE = major adverse cardiovascular events; PPM = permanent pacemaker; SNF = skilled nursing facility; TAVR = transcatheter aortic valve replacement; uOR = unadjusted odds ratio.

Values are %, OR (95% CI), or median (IQR). The **bold** values indicate statistical significance. The International Classification of Diseases-10th Revision (ICD-10) codes corresponding to each of the in-hospital outcomes were identified with the same process used to identify comorbidity codes (Supplemental Table 1).

a The multivariable regression model is adjusted for age, sex, race/ethnicity, insurance, hospital location and teaching status, bed size, region, type of admission, Elixhauser and Charlson comorbidity index scores, and relevant comorbidities (Supplemental Table 2).

b Cell counts <11 are not reportable (NR) per HCUP guidelines.

**Table 4:** In-Hospital Outcomes of TMVr and TMVR Stratified by Income

	TMVr				TMVR			
	High Income (n = 8,950)	Medium Income (n = 17,725)	Low Income (n = 7,145)	*P* Value	High Income (n = 1,000)	Medium Income (n = 2,070)	Low Income (n = 740)	*P* Value
Complications								
MACE								
%	3.2	3.0	2.6	0.58	7.5	7.7	6.8	0.93
uOR (95% CI)	Ref.	0.95 (0.69-1.33)	0.81 (0.54–1.21)	-	Ref.	1.03 (0.55-1.95)	0.89 (0.39–2.04)	-
aOR (95% CI)^a^	Ref.	0.99 (0.66-1.49)	0.93 (0.56–1.55)	-	Ref.	0.73 (0.33-1.63)	0.61 (0.21-1.79)	–
Death								
%	2.1	2.2	1.8	0.69	6.5	5.8	4.1	0.60
uOR (95% CI)	Ref.	1.07 (0.72-1.58)	0.88 (0.54-1.43)	–	Ref.	0.88 (0.44-1.76)	0.60 (0.23-1.62)	–
aOR (95% CI)^a^	Ref.	1.32 (0.80-2.18)	1.09 (0.59-2.03)	–	Ref.	0.51 (0.19-1.36)	0.29 (0.08-1.13)	–
Stroke								
%	1.2	1.2	0.9	0.68	NR^b^	2.4	3.4	0.31
uOR (95% CI)	Ref.	0.94 (0.54-1.62)	0.74 (0.37-1.48)	–	Ref.	2.45 (0.53-4.41)	3.46 (0.66-5.24)	–
aOR (95% CI)^a^	Ref.	0.82 (0.43-1.56)	0.88 (0.35-2.18)	–	Ref.	2.86 (0.58-4.80)	3.29 (0.50-5.67)	–
Acute kidney injury								
%	14.6	14.9	14.5	0.93	22.0	25.4	24.3	0.68
uOR (95% CI)	Ref.	1.02 (0.87-1.20)	0.99 (0.80-1.22)	–	Ref.	1.20 (0.79-1.83)	1.14 (0.67-1.93)	–
aOR (95% CI)^a^	Ref.	1.04 (0.85-1.27)	0.94 (0.72-1.24)	–	Ref.	1.10 (0.61-1.99)	0.95 (0.46-1.93)	–
Major bleeding								
%	11.3	10.0	10.2	0.34	29.0	30.7	23.0	0.20
uOR (95% CI)	Ref.	0.87 (0.72-1.05)	0.89 (0.71-1.13)	–	Ref.	1.08 (0.74-1.59)	0.73 (0.45-1.18)	–
aOR (95% CI)^a^	Ref.	0.85 (0.69-1.04)	0.97 (0.75-1.26)	–	Ref.	0.98 (0.62-1.55)	0.69 (0.39-1.19)	–
Vascular complications								
%	3.6	2.4	3.0	**0.03**	7.0	4.6	8.1	0.21
uOR (95% CI)	Ref.	**0.64 (0.46-0.90)**	0.82 (0.56-1.20)	–	Ref.	0.64 (0.31-1.30)	1.17 (0.52-2.63)	–
aOR (95% CI)^a^	Ref.	0.74 (0.55-1.01)	0.75 (0.48-1.17)	–	Ref.	0.56 (0.23-1.36)	1.16 (0.47-2.87)	–
PPM placement								
%	1.1	1.0	1.0	0.85	6.5	2.4	2.7	**0.03**
uOR (95% CI)	Ref.	0.86 (0.50-1.48)	0.94 (0.48-1.83)	–	Ref.	**0.36 (0.15-0.83)**	0.40 (0.13-1.26)	–
aOR (95% CI)^a^	Ref.	0.82 (0.47-1.44)	1.00 (0.48-2.07)	–	Ref.	0.29 (0.08-1.00)	0.48 (0.13-1.73)	–
Discharge disposition								
Routine	69.8	71.9	75.0	0.18	55.3	55.6	58.1	0.95
Transfer to short–term hospital	0.7	0.4	0.4		NR^b^	NR^b^	NR^b^	
Transfer to SNF or ICF	9.2	8.6	8.5		14.6	14.5	12.8	
Home health care	18.2	16.8	14.3		23.1	23.7	23.6	
Resource utilization								
LOS (d)	2 (1-4)	2 (1-4)	2 (1-4)	0.67	5 (2-11)	4 (2-9)	5 (2-10)	0.91
Hospital cost ($)	52,052 (35,760-84,857)	47,375 (32,931-78,096)	45,317 (31,677-74,934)	**<0.01**	71,495 (49,208-114,631)	65,011 (42,976-104,772)	62,870 (41,025-94,939)	**0.04**

aOR = adjusted odds ratio; ICF = intermediate care facility; LOS = length of stay; MACE = major adverse cardiovascular events; PPM = permanent pacemaker; SNF = skilled nursing facility; TMVr = transcatheter mitral valve repair; TMVR = transcatheter mitral valve replacement; uOR = unadjusted odds ratio.

Values are %, OR (95% CI), or median (IQR). The **bold** values indicate statistical significance. The International Classification of Diseases-10th Revision (ICD-10) codes corresponding to each of the in-hospital outcomes were identified with the same process used to identify comorbidity codes (Supplemental Table 1).

a The multivariable regression model is adjusted for age, sex, race/ethnicity, insurance, hospital location and teaching status, bed size, region, type of admission, Elixhauser and Charlson comorbidity index scores, and relevant comorbidities (Supplemental Table 2).

b Cell counts <11 are not reportable (NR) per HCUP guidelines.

## Discussion

We report economic disparities in the utilization and outcomes of SHD interventions in the United States. This analysis of the large, nationally representative NIS database produced several novel findings: 1) common SHD interventions are performed less frequently in low- and medium-income patients compared with high-income patients; 2) from 2016 through 2020, the use of SHD interventions increased significantly in all 3 income groups; 3) significant differences exist in demographic, hospital, and clinical characteristics among high-, medium-, and low-income patients who undergo SHD interventions in contemporary U.S. practice; and 4) in-hospital mortality, morbidity, and LOS of common SHD interventions were comparable across the 3 income groups, but the cost was higher in high-income patients.

### Disparities in SHD intervention use

Our study found lower utilization of SHD interventions in patients with low and medium income compared with high income. This aligns with a prior, smaller study evaluating socioeconomic disparities in access to TAVR, which found that for each $1,000 decrease in median household income, the number of TAVR procedures performed per 100,000 Medicare beneficiaries was 0.2% (95% CI: 0.1%-0.4%) lower (*P* < 0.01).^14^ This finding is further supported by our prior study, which found that patients ≥80 years of age who underwent TAVR were more likely to have a household income in the highest quartile.^15^

Disparities in utilization of SHD interventions across income groups are likely multifactorial, including financial limitations, low healthcare literacy, subpar patient-provider communication, provider bias and hesitancy due to concerns about medication noncompliance and adherence to follow-up appointments, and inequitable access to advanced SHD procedures among low-income individuals. The increased likelihood of low-income patients being female, Black, or Hispanic in our study likely also contributes to the gap in utilization rates, as prior studies have demonstrated significant underrepresentation of women as well as Black and Hispanic patients compared to White patients in SHD interventions.^6^^,^^7^^,^^16^ Finally, geographic differences in care may also contribute to the limited access to SHD interventions, as low-income patients were more likely to receive care in rural hospitals with small bed sizes compared with high-income patients. Centers of SHD care are not uniformly distributed geographically; instead they are clustered in large cities with large academic medical centers often serving affluent populations. This disparity has been present since the dawn of SHD interventions, with Nathan et al.^17^ reporting that, during the initial growth phase of TAVR programs in the United States, hospitals serving wealthier patients were more likely to start TAVR programs. This pattern of growth has led to inequities in the dispersion of TAVR, with lower rates in poorer communities.^17^

Multifaceted implementation strategies are needed to attenuate economic disparities in the utilization of SHD interventions. Providers should undergo training to recognize and address implicit biases in the care of patients with SHD, as these unconscious biases often influence clinical decisions inadvertently, perpetuating disparities in SHD interventions.^18^ Continued work to increase the diversity of the physician workforce is needed to mitigate socioeconomic disparities in SHD interventions, as prior data have shown racial concordance between patients and providers to be associated with improved healthcare outcomes, driven by more effective communication and shared decision-making.^19^^,^^20^ “Safety net hospitals,” which predominantly serve low- and middle-income patients, often face significant resource limitations in SHD care, which are further aggravated by geographical disparities in access to SHD interventions.^21^ Improving access to SHD interventions at safety net hospitals is therefore essential to addressing disparities in SHD care.^21^ This improved access will require increased resources through federal, state, and local funding and grants, promoting peer-to-peer training in SHD interventions for providers in underserved communities, and involving safety net hospitals in clinical trials of SHD therapies.^21^ Improved resources in safety net hospitals may also attract interventional cardiologists trained in SHD interventions, thus making these treatments more accessible. Governmental and grant funding to facilitate the acquisition of advanced interventional and imaging technologies used in SHD interventions will reduce barriers to SHD care and cultivate training pathways for SHD intervention and imaging in safety net hospital communities.^21^^,^^22^ Finally, increasing healthcare literacy in communities with low socioeconomic status through educational programs, healthcare advocacy, and community outreach may further reduce disparities in access to SHD interventions.

### Temporal trends in SHD interventions

Despite the underutilization of SHD interventions among low- and medium-income groups, our study found a temporal increase in SHD interventions performed in all 3 income groups. These increases are congruent with a prior study evaluating trends in SHD interventions that noted that the number of TAVR, LAAO, and mitral transcatheter edge-to-edge repair procedures increased significantly from 2016 to 2020.^23^ Likewise, the number of TMVR procedures increased significantly in all racial/ethnic and sex groups from 2016 to 2020.^15^^,^^24^ Increased SHD interventions across all income groups can be attributed to: 1) improved safety, efficacy, and feasibility of catheter-based SHD interventions, compared to surgical methods, as a result of rapid advancements in device technology and techniques as well as enhanced operator experience;^25^^,^^26^ and 2) broadened indications for SHD interventions.^3^^,^^26^

### In-hospital outcomes, LOS, and costs

Our study found similar in-hospital outcomes among patients undergoing SHD interventions across all income groups. These reassuring data are in line with prior studies showing comparable adjusted in-hospital outcomes of LAAO between income quartiles.^27^ Nonetheless, further studies evaluating the long-term outcomes of SHD interventions in different income groups are still warranted.

Hospitalization costs were significantly higher among high-income patients compared to low- and medium-income patients undergoing SHD interventions. This cost gradient is congruent with work by Sparrow et al, ^27^ which reported that as income quartile increased, LAAO hospitalization costs increased significantly. Regional variations may contribute to the discrepancy in hospitalization costs, as prior studies have demonstrated lower hospitalization costs in the Midwest and South regions, where incomes were lower among SHD intervention patients in our study, and higher hospitalization costs in the Northeast and West, where incomes were higher among SHD intervention patients.^28^

### Study Limitations

Our study has several important limitations. First, in a retrospective NIS study using administrative claims codes, incorrect coding could lead to inaccurate data. Second, the retrospective nature of the study makes it subject to inherent selection bias. Third, detailed baseline and procedural characteristics such as echocardiographic findings, access site, and peri-procedural medications were unavailable, which can lead to unmeasured bias. Fourth, validated risk stratification scores, such as the Society of Thoracic Surgeons score, are not captured by the NIS, limiting patient risk assessment. Fifth, some variables (eg, insurance) had small categories, and real differences between the categories may not be statistically significant due to small sample sizes. Sixth, the NIS allows detailed assessment of in-hospital outcomes but does not include long-term clinical outcomes beyond discharge. Studies exploring long-term economic disparities in the outcomes of SHD interventions are still needed. Seventh, other dimensions of disparity, such as residential instability and education level, are not available in the NIS and require further research.

Despite these limitations, this study adds meaningfully to the literature by describing contemporary economic disparities in the utilization and outcomes of SHD interventions. The NIS is well validated for outcomes studies like this one and undergoes serial data accuracy checks and quality control. In addition, the NIS data are geographically diverse, including a nationally representative sample of centers and operators, and hence reliably reflect real-world practice and outcomes.

## Conclusions

Economic disparities exist in the utilization of SHD interventions in the United States. Nonetheless, adjusted in-hospital outcomes were comparable among high-, medium-, and low-income patients. Multifaceted implementation strategies are needed to attenuate these utilization disparities.

PERSPECTIVES **COMPETENCY IN SYSTEMS-BASED PRACTICE:** Common SHD interventions are performed less frequently in low- and medium-income patients compared with high-income patients in the United States. Low- and medium-income patients undergoing SHD interventions have distinctive demographic and clinical risk profiles compared with high-income patients. Nonetheless, adjusted in-hospital outcomes and the LOS of SHD interventions are comparable across the 3 income groups.

**TRANSLATIONAL OUTLOOK:** Further studies are warranted to clarify the reasons for economic disparities in the utilization of SHD interventions in the United States and to guide policy initiatives to achieve equity.

## Funding support and author disclosures

This work was supported by the Department of Cardiovascular Medicine at Mayo Clinic in Rochester, Minnesota, USA. Dr Goldsweig reports consulting for Philips and Inari Medical; and speaking for Philips and Edwards Lifesciences. All other authors have reported that they have no relationships relevant to the contents of this paper to disclose.
