# Supplementary material for: Economic Disparities in Utilization and Outcomes of Structural Heart Disease Interventions in the United States
Source: JACC Adv. 2024 Jul 3;3(7):101034. doi: 10.1016/j.jacadv.2024.101034 (PMC11312775; doi:10.1016/j.jacadv.2024.101034)
Supplement: Supplemental Data [file mmc1.docx]

**Supplemental Table 1.** ICD-10 diagnosis (CM) and procedure (PCS) codes

| **SHD procedures** | **ICD-10 PCS codes** |
| --- | --- |
| TAVR | 02RF37H, 02RF38H, 02RF3JH, 02RF3KH, X2RF332, 02RF37Z, 02RF38Z, 02RF3JZ, 02RF3KZ |
| Percutaneous LAAO | 02L73DK |
| TMVr | 02UG3JH, 02UG3JZ, 02QG3ZE, 02QG3ZZ |
| TMVR | 02RG37H, 02RG38H, 02RG3JH, 02RG3KH, 02RG37Z, 02RG38Z, 02RG3JZ, 02RG3KZ |

| **Baseline characteristics** | **ICD-10 CM codes** |
| --- | --- |
| **Comorbidities** | |
| Diabetes mellitus | E10.0, E10.1, E10.9, E11.0, E11.1, E11.9, E12.0, E12.1, E12.9, E13.0, E13.1, E13.9, E14.0, E14.1, E14.9, E10.2-E10.8, E11.2-E11.8, E12.2-E12.8, E13.2-E13.8, E14.2-E14.8 |
| Hypertension | I10.x, I11.x-I13.x, I15.x |
| Dyslipidemia | E78.x |
| Nicotine/tobacco use | F17.x, Z72.0, Z87.891 |
| Alcohol abuse | F10, E52, G62.1, I42.6, K29.2, K70.0, K70.3, K70.9, T51.x, Z50.2, Z71.4, Z72.1 |
| Drug abuse | F11.x-F16.x, F18.x, F19.x,  Z71.5. Z72.2 |
| Obesity | E66.x |
| Coronary artery disease | I25.x |
| Peripheral vascular disease | I70.x, I71.x, I73.1, I73.8, I73.9, I77.1, I79.0, I79.2, K55.1, K55.8, K55.9, Z95.8, Z95.9 |
| Congestive heart failure | I09.9, I11.0, I13.0, I13.2, I25.5, I42.0, 142.5-I42.9, I43.x, I50.x, P29.0 |
| Renal failure | I12.0, I13.1, N18.x, N19.x, N25.0, Z49.0-Z49.2, Z94.0, Z199.2 |
| Dialysis dependent | Z99.2 |
| Liver disease | B18.x, I85.x, I86.4, I98.2, K70.x, K71.1, K71.3-K71.5, K71.7, K72.x-K74.x, K76.0, K76.2-K76.9. Z94.4 |
| Chronic pulmonary disease | I27.8, 127.9, J40.x-J47.x, J60.x-J67.x, J68.4, J70.1, J70.3 |
| Obstructive sleep apnea | G47.33 |
| Coagulopathy | D65-D68.x, D69.1, D69.3-D69.6 |
| Cancer | C0x.x, C1x.x, C2x.x, C30.x, C31.x, C32.x, C33.x, C34.x, C37.x, C38.x, C39.x, C40.x, C41.x, C43.x, C45.x, C46.x, C47.x, C48.x, C49.x, C50, C51-58.x, C60-63.x, C76.x, C80.1, C81.x, C82.x, C83.x, C84.x, C85.x, C88.x, C9x.x |
| Malnutrition | E43, E44.x, E45, E46 |
| Dementia | F01.x, F02.x, F03.x, F04, F05, F06.1, F06.8, G13.2, G13.8, G30.x, G31.0x, G31.1, G31.2, G91.4, G94, R41.81, R54 |
| Depression | F20.4, F31.3-F31.5, F32.x, F33.x, F34.1, F41.2, F43.2 |
| **Previous history** | |
| Myocardial infarction | I25.2 |
| Stroke/TIA | Z86.73 |
| Cardiac arrest | Z86.74 |
| PCI | Z98.61, Z95.5 |
| CABG | Z95.1 |
| ICD | Z95.810 |
| PPM | Z95.0 |

| **In-hospital outcomes** | **ICD-10 CM/PCS codes** |
| --- | --- |
| Stroke | I63.x, I67.81, I67.82, G45.x, G46.x, H34.0x, H34.1x, H34.2x, I60.x, I61.x, I62.x, I97.820, I97.810 |
| Acute kidney injury | N17.x, N99.0 |
| Major bleeding | I97.610, I97.410, I97.618, I97.418, L76.22, L76.02, I97.51, L76.12, K92.0, K92.1, K92.2, K91.841, K91.62, R31.0, N99.821, N99.62, R04.x, J95.831, J95.62, R58, D62 |
| Vascular complications | I77.0, I72.x, L76.32, L76.02, I97.630, I97.410, I97.638, I97.418, K66.1, I26.92, I26.93, I26.94, I26.99, I26.02, I26.09, I82.4x, T81.718A, T81.719A, T81.72XA |
| PPM placement | 0JH634Z, 0JH635Z, 0JH636Z, 0JH63PZ, 0JH834Z, 0JH835Z, 0JH836Z, 0JH83PZ, 0JH604Z, 0JH605Z, 0JH606Z, 0JH60PZ, 0JH804Z, 0JH805Z, 0JH806Z, 0JH80PZ |
| Cardiac tamponade | I31.4 |

**Supplemental Table 2.** Variables used in the multivariable regression analysis to compute adjusted odds of in-hospital outcomes

| **Demographic characteristics** |
| --- |
| Age |
| Biological sex |
| Race/ethnicity |
| Insurance |
| **Hospital characteristics** |
| Location/teaching status |
| Bed size |
| Region |
| Elective admission |
| Weekend admission |
| **Clinical characteristics** |
| Elixhauser comorbidity index |
| Charlson comorbidity index |
| Diabetes mellitus |
| Hypertension |
| Dyslipidemia |
| Nicotine/tobacco use |
| Alcohol abuse |
| Drug abuse |
| Obesity |
| Coronary artery disease |
| Peripheral vascular disease |
| Congestive heart failure |
| Renal failure |
| Dialysis dependent |
| Liver disease |
| Chronic pulmonary disease |
| Obstructive sleep apnea |
| Coagulopathy |
| Cancer |
| Malnutrition |
| Dementia |
| Depression |
| Previous history of myocardial infarction |
| Previous history of stroke/TIA |
| Previous history of cardiac arrest |
| Previous history of PCI |
| Previous history of CABG |
| Previous history of ICD |
| Previous history of PPM |

**Supplemental Table 3.** Modification of the effect of income on in-hospital outcomes of TAVR by race

|  | **White** | | | **Non-White** | | |  |
| --- | --- | --- | --- | --- | --- | --- | --- |
|  | High income (Ref.) | Low income | aOR (95% CI)^a^  or *p* | High income  (Ref.) | Low income | aOR (95% CI)^a^ or *p* | Interaction *p* |
| **Complications** |  |  |  |  |  |  |  |
| MACE | 3.4 | 3.6 | 1.19 (0.99–1.41) | 3.8 | 3.5 | 1.12 (0.76–1.65) | 0.53 |
| Death | 1.2 | 1.5 | 1.33 (0.97–1.72) | 1.4 | 1.1 | 0.83 (0.45–1.54) | 0.16 |
| Stroke | 2.4 | 2.4 | 1.12 (0.90–1.40) | 2.4 | 2.5 | 1.26 (0.76–2.09) | 0.82 |
| Acute kidney injury | 9.0 | 9.7 | 1.11 (0.98–1.25) | 11.4 | 12.3 | 1.34 (0.95–1.71) | 0.18 |
| Major bleeding | 14.3 | 14.5 | 1.01 (0.90–1.13) | 14.9 | 16.0 | 1.08 (0.89–1.30) | 0.93 |
| Vascular complications | 4.8 | 4.2 | 0.95 (0.82–1.10) | 4.7 | 5.2 | 1.01 (0.75–1.35) | 0.25 |
| PPM placement | 9.0 | 7.2 | 0.84 (0.74–1.02) | 7.5 | 7.8 | 1.13 (0.87–1.47) | 0.10 |
| Cardiac tamponade | 0.8 | 0.6 | 0.76 (0.54–1.07) | 0.9 | 0.3 | 0.58 (0.26–1.29) | 0.62 |
| **Non-home discharge** | 33.6 | 29.6 | 0.91 (0.84–1.02) | 34.7 | 34.8 | 0.97 (0.82–1.15) | 0.13 |
| **Resource utilization** |  |  |  |  |  |  |  |
| LOS (days) | 2 (1-4) | 2 (1-4) | 0.31 | 2 (1-5) | 2 (1-6) | 0.20 | 0.26 |
| Hospital cost ($) | 56,528 (39,729-85,389) | 50,001 (35,464-76,956) | **<0.01** | 55,503 (38,884-92,581) | 53,152 (36,147-85,426) | **<0.01** | 0.14 |

Data presented as %, median (IQR), or as indicated in table. The bold values indicate statistical significance.

^a^The multivariable regression model is adjusted for age, sex, race/ethnicity, insurance, hospital location and teaching status, bed size, region, type of admission, Elixhauser and Charlson comorbidity index scores, and relevant comorbidities (*Supplemental* *Table 2*).

**Supplemental Table 4.** Modification of the effect of income on in-hospital outcomes of LAAO by race

|  | **White** | | | **Non-White** | | |  |
| --- | --- | --- | --- | --- | --- | --- | --- |
|  | High income (Ref.) | Low income | aOR (95% CI)^a^  or *p* | High income  (Ref.) | Low income | aOR (95% CI)^a^  or *p* | Interaction *p* |
| **Complications** |  |  |  |  |  |  |  |
| MACE | 0.7 | 0.7 | 1.09 (0.57–2.08) | 1.1 | 1.4 | 1.46 (0.47–2.95) | 0.66 |
| Death | NR^b^ | 0.2 | 2.11 (0.79–6.78) | NR^b^ | 0.5 | 2.57 (0.75–8.78) | 0.81 |
| Stroke | 0.7 | 0.6 | 0.82 (0.38–1.75) | 0.9 | 1.1 | 1.68 (0.45–2.39) | 0.49 |
| Acute kidney injury | 2.4 | 1.9 | 0.85 (0.58–1.25) | 2.4 | 4.1 | 1.09 (0.43–2.73) | 0.41 |
| Major bleeding | 5.9 | 5.7 | 0.95 (0.73–1.23) | 6.2 | 8.6 | 1.38 (0.83–2.29) | 0.51 |
| Vascular complications | 1.1 | 1.2 | 1.48 (0.88–2.49) | NR^b^ | 1.5 | 1.53 (0.65–2.58) | 0.26 |
| PPM placement | 1.3 | 1.3 | 0.97 (0.57–1.63) | 0.6 | 1.8 | 1.64 (0.48–5.66) | 0.24 |
| Cardiac tamponade | 0.6 | 0.7 | 1.14 (0.57–2.29) | 0.5 | 0.9 | 1.54 (0.77–2.43) | 0.31 |
| **Non-home discharge** | 7.0 | 7.3 | 1.19 (0.93–1.52) | 7.6 | 10.0 | 1.36 (0.84–2.22) | 0.56 |
| **Resource utilization** |  |  |  |  |  |  |  |
| LOS (days) | 1 (1-1) | 1 (1-1) | 0.34 | 1 (1-1) | 1 (1-1) | 0.72 | 0.46 |
| Hospital cost ($) | 30,237 (21,700-46,091) | 26,992 (19,355-38,807) | **<0.01** | 29,775 (21,326-48,557) | 27,695 (19,895-40,453) | **<0.01** | 0.86 |

Data presented as %, median (IQR), or as indicated in table. The bold values indicate statistical significance.

^a^The multivariable regression model is adjusted for age, sex, race/ethnicity, insurance, hospital location and teaching status, bed size, region, type of admission, Elixhauser and Charlson comorbidity index scores, and relevant comorbidities (*Supplemental* *Table 2*).

^b^Cell counts <11 are not reportable (NR) per HCUP guidelines.

**Supplemental Table 5.** Modification of the effect of income on in-hospital outcomes of TMVr by race

|  | **White** | | | **Non-White** | | |  |
| --- | --- | --- | --- | --- | --- | --- | --- |
|  | High income (Ref.) | Low income | aOR (95% CI)^a^  or *p* | High income  (Ref.) | Low income | aOR (95% CI)^a^  or *p* | Interaction *p* |
| **Complications** |  |  |  |  |  |  |  |
| MACE | 3.2 | 2.4 | 1.02 (0.59–1.77) | 2.9 | 3.1 | 1.89 (0.58–6.12) | 0.19 |
| Death | 1.9 | 1.5 | 1.06 (0.53–2.11) | 2.9 | 2.7 | 1.33 (0.47–3.79) | 0.77 |
| Stroke | 1.4 | 1.1 | 1.57 (0.58–4.23) | NR^b^ | NR^b^ | 1.65 (0.48–4.52) | 0.89 |
| Acute kidney injury | 13.7 | 13.6 | 0.94 (0.67–1.34) | 19.3 | 16.6 | 0.70 (0.40–1.23) | 0.23 |
| Major bleeding | 10.8 | 11.1 | 1.21 (0.89–1.64) | 13.8 | 8.0 | 0.97 (0.75–1.35) | 0.35 |
| Vascular complications | 3.8 | 3.1 | 0.69 (0.41–1.16) | 2.9 | 2.9 | 0.56 (0.16–1.89) | 0.85 |
| PPM placement | 1.1 | 0.9 | 1.15 (0.45–2.93) | 1.5 | 1.4 | 0.86 (0.40–2.16) | 0.71 |
| **Non-home discharge** | 29.2 | 20.5 | 0.83 (0.57–1.13) | 25.8 | 31.4 | 1.33 (0.82–2.24) | 0.18 |
| **Resource utilization** |  |  |  |  |  |  |  |
| LOS (days) | 2 (1-4) | 1 (1-3) | 0.20 | 2 (1-4) | 2 (1-5) | 0.42 | 0.37 |
| Hospital cost ($) | 52,333 (36,202-84,981) | 43,655 (31,274-71,621) | **<0.01** | 51,060 (34,587-85,357) | 45,376 (31,591-74,663) | **<0.01** | 0.36 |

Data presented as %, median (IQR), or as indicated in table. The bold values indicate statistical significance.

^a^The multivariable regression model is adjusted for age, sex, race/ethnicity, insurance, hospital location and teaching status, bed size, region, type of admission, Elixhauser and Charlson comorbidity index scores, and relevant comorbidities (*Supplemental* *Table 2*).

^b^Cell counts <11 are not reportable (NR) per HCUP guidelines.

**Supplemental Table 6.** Modification of the effect of income on in-hospital outcomes of TMVR by race

|  | **White** | | | **Non-White** | | |  |
| --- | --- | --- | --- | --- | --- | --- | --- |
|  | High income (Ref.) | Low income | aOR (95% CI)^a^  or *p* | High income  (Ref.) | Low income | aOR (95% CI)^a^  or *p* | Interaction *p* |
| **Complications** |  |  |  |  |  |  |  |
| MACE | 7.1 | 6.4 | 0.62 (0.25–1.78) | 9.1 | 7.9 | 0.52 (0.23–1.69) | 0.79 |
| Death | 5.8 | 3.6 | 0.39 (0.18–1.25) | 9.1 | NR^b^ | 0.25 (0.13–1.37) | 0.59 |
| Stroke | NR^b^ | 3.6 | 3.15 (0.71–4.82) | 0 | NR^b^ | 3.62 (0.69–5.02) | 0.74 |
| Acute kidney injury | 20.5 | 20.0 | 0.74 (0.29–1.88) | 27.3 | 36.8 | 0.87 (0.44–1.91) | 0.86 |
| Major bleeding | 28.8 | 22.7 | 0.73 (0.37–1.43) | 29.5 | 23.7 | 0.75 (0.41–1.57) | 0.71 |
| Vascular complications | 6.4 | 8.2 | 0.86 (0.32–2.33) | 9.1 | 7.9 | 0.72 (0.25–2.11) | 0.89 |
| PPM placement | 5.8 | 2.7 | 0.40 (0.13–2.16) | 9.1 | NR^b^ | 0.42 (0.17–2.34) | 0.47 |
| **Non-home discharge** | 43.2 | 35.8 | 1.13 (0.53–2.39) | 32.5 | 50.0 | 1.67 (0.85–2.72) | 0.10 |
| **Resource utilization** |  |  |  |  |  |  |  |
| LOS (days) | 5 (1-11) | 4 (2-9) | 0.93 | 5 (3-11) | 7 (2-10) | 0.65 | 0.32 |
| Hospital cost ($) | 71,495 (49,485-120,096) | 65,723 (41,184-100,027) | **<0.01** | 71,298 (47,985-94,970) | 59,999 (40,497-84,923) | **<0.01** | 0.90 |

Data presented as %, median (IQR), or as indicated in table. The bold values indicate statistical significance.

^a^The multivariable regression model is adjusted for age, sex, race/ethnicity, insurance, hospital location and teaching status, bed size, region, type of admission, Elixhauser and Charlson comorbidity index scores, and relevant comorbidities (*Supplemental* *Table 2*).

^b^Cell counts <11 are not reportable (NR) per HCUP guidelines.
